# Dietary Genistein Influences Number of Acetylcholine Receptors in Female Diabetic Jejunum

**DOI:** 10.1155/2017/3568146

**Published:** 2017-08-01

**Authors:** Sydney Schacht, Faisal Masood, Shawn Catmull, Robert Dolan, RussL Altabtabaee, Wade Grow, Layla Al-Nakkash

**Affiliations:** ^1^Department of Physiology, Midwestern University, 19555 N. 59th Avenue, Glendale, AZ 85308, USA; ^2^Department of Anatomy, Arizona College of Osteopathic Medicine, Midwestern University, 19555 N. 59th Avenue, Glendale, AZ 85308, USA

## Abstract

**Background:**

Intestinal dysfunction in the *ob/ob* mouse model of diabetes mimics that seen clinically.

**Methods:**

We determined the effects of a 4-week genistein diet (600 mg genistein/kg food) on intestinal function (contractility, morphology, AChR, and motility) in female *ob/ob* and lean mice.

**Results:**

Contractility of the jejunum in response to incrementally increasing concentrations of KCl was comparable in *ob/ob* females and lean controls regardless of a genistein-diet. There were no changes in the wall thickness measured. We assessed the number of clusters of AChR in the jejunum wall; AChR were decreased by 48% in *ob/ob* mice versus leans, and the genistein diet reversed this. In utilizing a video-imaging system to evaluate gastrointestinal motility, we determined that the distance between consecutive contractile events was significantly increased by 1.87-fold in *ob/ob* mice versus leans, and the genistein diet was without effect.

**Conclusions:**

These data suggest that slowed intestinal transit in the diabetic *ob/ob* mouse may be due in part to decreased AChR and decreased contraction events occurring per unit time. A genistein diet rescues the number of AChR to levels of leans yet did not change the number of contractile events. Feeding *ob/ob* mice a genistein-rich diet has potential therapeutic benefits towards improving the debilitating diabetes-related gastrointestinal dysfunction.

## 1. Background

Slowed gastrointestinal transit and delayed gastric emptying time (gastroparesis) are known clinical complications associated with diabetes and obesity; in fact, gastroparesis is seen in ~30% of diabetic patients [1–4]. The *ob/ob* leptin-deficient mouse is a commonly utilized murine model of diabetes and obesity that closely mimics the gastrointestinal dysfunction seen clinically, including slower gastrointestinal transit and delayed gastric emptying time [2, 5].

It is predicted that the pathogenesis of diabetic-associated slowed intestinal transit will involve abnormalities in intrinsic and extrinsic nervous systems and/or smooth muscle cells within the intestinal tract wall, each of which play interconnected and vital roles in the pattern of intestinal contractile activity. Intestinal smooth muscle contraction is elicited as a consequence of a number of regulated events occurring in sequence. The mechanism associated with the etiology of gastroparesis has been widely studied, and pathways that are dysregulated in gastric smooth muscle which contribute towards the slowed gastric emptying include reduced myosin light chain phosphatase inhibition [6]. However, in the *ob/ob* mouse jejunum, the pathways that likely contribute towards the intestinal dysfunction (i.e., slowed intestinal transit) remain unclear, and furthermore, no studies have assessed the contribution that dietary genistein may play to ameliorate such pathology. Genistein is a naturally occurring phytoestrogen found in soy [7]. Serum levels of genistein can reach micromolar concentrations [8]. Mice, consuming a genistein diet (600 mg genistein/kg diet), can generate serum genistein levels of 2–4 *μ*M [9] which is comparable to the serum levels obtained in humans consuming a soy milk diet [10]. We have previously shown that *ob/ob* mice have reduced basal transepithelial chloride secretory function across jejunum tissue [11]. More recently, we have established that consumption of a genistein diet (600 mg genistein/kg diet) for 4 weeks reverses this deficit in jejunal chloride secretion in the *ob/ob* mouse [12].

The mechanisms involved in mediating dysfunctional gastrointestinal transit in diabetic mouse models are complex and less well understood. In this study, we provide an assessment of the effects of a genistein diet upon jejunum motility and contractility, jejunum wall thickness, and a quantification of acetylcholine receptors (AChR) in *ob/ob* mouse jejunum. We hypothesized that the diabetic obese *ob/ob* mouse has dysfunctional jejunum motility, along with downregulation of AChR and that these deficiencies could be ameliorated by the administration of a genistein diet for a period of 4 weeks.

## 2. Methods

Female ob/ob and lean littermate C57BL/6J mice aged 5 weeks were purchased from Jackson Laboratory (Bar Harbor, ME) and housed in an animal care facility with a 12 : 12-hour light-dark cycle. Mice were randomly assigned to one of the two diet groups: fed either a standard rodent chow (std) or fed a genistein-containing diet (Gen). The genistein-containing diet (Gen) was purchase from Dyets Inc. (Bethlehem, PA) and contained 600 mg genistein/kg diet. The composition of the genistein diet has been published previously [9, 13, 14]. Food and water were provided ad libitum. Body weight and general health were monitored weekly for the duration of the 4-week diet study period. At ~10*–*12 weeks of age, mice (ob/ob and lean) were asphyxiated in an atmosphere of 100% CO_2_, followed by surgical pneumothorax. Animal care was conducted in accordance with established guidelines, and all protocols were approved by the Midwestern University Institutional Animal Care and Use Committee.

### 2.1. Histology and Morphology: H&E Staining

Freshly isolated pieces of the jejunum were embedded and flashed frozen in Optimal Cutting Temperature compound (OCT, Tissue-Tek, Torrance, CA). For hematoxylin and eosin (H&E) staining, frozen sliced sections of the jejunum (10 *μ*m) were stained with a standard protocol, prior to performing the morphometric analyses to evaluate basic histological measurements. In brief, sections were exposed to the following wash protocols: hematoxylin 30 s, water rinse 10 s, Scott's solution 5 s, water rinse 10 s, 95% ethanol 5 s, eosin 15 s, rinse with 95% ethanol 10 s, and then 100% ethanol 10 s, followed by xylene 15 s. Inner circular smooth muscle wall thickness (and the number of nuclei within this region), outer longitudinal wall thickness (and the number of nuclei within this region), and total wall thickness were measured using Axiovision (Carl Zeiss), from images of H&E-stained jejunum sections. All images were taken at 10x magnification. Averages of measurements were taken from 5 separate slices per frozen section of jejunum (i.e., per mouse), and data are presented as the average of multiple mice per group.

### 2.2. Acetylcholine Receptor (AChR) Immunofluorescence

AChRs were visualized as described previously [15], with a fluorescence microscope by the binding of *α*-bungarotoxin conjugated to tetramethylrhodamine. Sections of the jejunum (10 *μ*m) were incubated in the toxin-containing medium for 30 min at 37°C to label AChRs. Sections were then rinsed twice with room temperature phosphate-buffered saline (PBS), fixed for 10 min with room temperature 2% paraformaldehyde in PBS, rinsed twice with room temperature PBS, dehydrated in cold methanol for 5 min at −20°C, and mounted in buffered glycerol containing paraphenylenediamine. Bright clusters of AChRs were observed, and average clusters per field of view were determined for 3-4 randomly chosen fields per animal. Fluorescence and phase contrast images were captured from the Olympus fluorescence microscope with the Magnafire Digital Camera, using a 20x objective.

### 2.3. Contractility Measures

In a subset of mice, freshly isolated jejunum rings were prepared for measures of isometric tension. Each mouse yielded 2 rings of jejunum tissue. All segments of the jejunum were subjected to the same procedures, and data were averaged and considered representative for that animal, considered *n* = 1. Isometric tension was measured using standard isolated tissue procedures. Briefly, jejunum rings were mounted between 2 stainless steel wires that were passed through the lumen of each jejunum ring, with wires connected to a force transducer (159901A, Radnoti, Monrovia, CA). Jejunum rings were submerged in a 15 ml bath containing a Krebs bicarbonate ringer, in mM: 115 NaCl, 25 NaHCO_3_, 5 KCl, 1.2 MgCl_2_, and 1.2 CaCl_2_ (pH 7.4), and equilibrated at 37°C with a 95% oxygen/5% carbon dioxide gas mixture. Jejunum rings were stretched and maintained at ~0.275 g resting tension and equilibrated for ~40 min with frequent bath changes. Dose-response curves of tension generated by cumulative addition of increasing concentrations of potassium chloride were obtained (with steady-state contractile responses achieved at each dose, 0–100 mM). Tension development was continuously recorded and collected by PowerLab with ChartPro computerized data acquisition system software (AD Instruments Inc., Colorado Springs, CO).

### 2.4. Gastrointestinal Motility Measures

A freshly isolated segment of the jejunum (3–5 cm) was placed in ice-cold Krebs buffer (in mM: 121 NaCl, 5.9 KCl, 2.5 CaCl_2_, 1.2 MgCl_2_, 25 NaHCO_3_, 1.2 NaH_2_PO_4_, and 8 glucose), oxygenated with 95% O_2_/5% CO_2_. Mesentery was cleaned from the outer wall. The tissue was then pinned in place and allowed to equilibrate for 20 minutes in a continuously perfused organ bath with warmed (37°C) and oxygenated (95%/5% O_2_/CO_2_) Kreb's buffer. The gastrointestinal motility monitoring system (GIMM, Catamount, Vermont) illuminates the intestinal segment from beneath. A digital video camera interfaced with a computer is positioned above the intestinal segment. Ensure that both the light illumination source and GIMM software are turned on. A series of 5–9- and 60-second recordings of intestinal motility were taken. Each captured video of motility is converted into a spatiotemporal map with the GIMM software, and the resulting maps were analyzed in Image J (NIH). Measures were made of velocity (the slope of lines), total distance (the length of lines) and total time (based on length of line), number of events in a given time period, and the distance between consecutive active regions (dark regions are dilated/relaxed tissue and light regions are constricted/contracted tissue). Five measures were made of each parameter per mouse, and the average for that mouse was used.

### 2.5. Chemicals

Antibodies for AChR-bungarotoxin conjugated to tetramethylrhodamine were purchased from Molecular Probes (Life Technologies, Carlsbad, CA). All other chemicals were obtained from Sigma-Aldrich (St. Louis, MO).

### 2.6. Statistics

Data are expressed as mean ± SEM. Numbers in parentheses represent the numbers of tissues used from separate individual mice. Unpaired *t*-tests were performed using GraphPad (San Diego, CA), and *P* < 0.05 was considered statistically significant.

## 3. Results

The *ob/ob* female mice fed a standard diet (51.50 ± 1.55 g, *n* = 23) were 2.2-fold heavier than lean counterparts (23.67 ± 0.43 g, *n* = 25), and the genistein diet induced a 12% weight loss in *ob/ob* females (45.57 ± 0.93 g, *n* = 33).

### 3.1. Contractility

**Figure 1:**
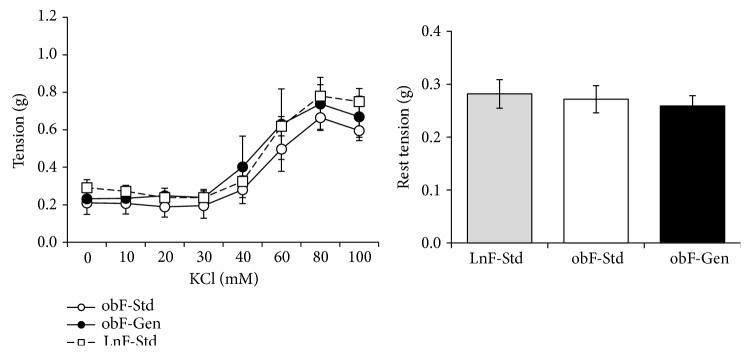
Effect of genistein on jejunum contractility. Average tissue contractility in females determined from freshly isolated segments of jejunum in response to incrementally increasing concentrations of KCl (0–100 mM) in the tissue bath. Lean mice fed standard diet (open square), *ob/ob* mice fed standard diet (open circle), and *ob/ob* mice fed genistein diet (solid circle). Average tissue rest tension in females. Average rest tension (g) of the freshly isolated segments of jejunum at the start of the contractility experiments: lean mice fed standard diet (gray bar), *ob/ob* mice fed standard diet (open bar), and *ob/ob* mice fed genistein diet (solid bar). Values are mean ± SEM (*n* = 6–14).

Tension generated in freshly isolated segments of the jejunum was measured for each group of mice (Figure 1). Contractility increased in all female groups as a function of incrementally increasing the dose of potassium chloride (10–100 mM) in the jejunum (Figure 1). The maximum tension generated with 100 mM KCl was comparable in *ob/ob* standard-fed mice (0.60 ± 0.05 g, *n* = 6) versus lean controls (0.75 ± 0.07 g, *n* = 12). There was no effect of the genistein diet on the maximum tension generated (0.60 ± 0.11 g, *n* = 9). Resting tensions were not different between any of the groups in females (Figure 1: lean standard fed = 0.28 ± 0.03 g, *n* = 12; *ob/ob* standard fed = 0.27 ± 0.02 g, *n* = 9; and *ob/ob* genistein fed = 0.26 ± 0.02 g, *n* = 9). The wet weight of each jejunum segment was not different between the groups; lean standard fed = 41.16 ± 2.75 mg (*n* = 12), *ob/ob* standard fed = 49.23 ± 3.44 mg (*n* = 6), and *ob/ob* genistein fed = 49.79 ± 3.36 m (*n* = 9).

### 3.2. Jejunum Morphology

**Figure 2:**
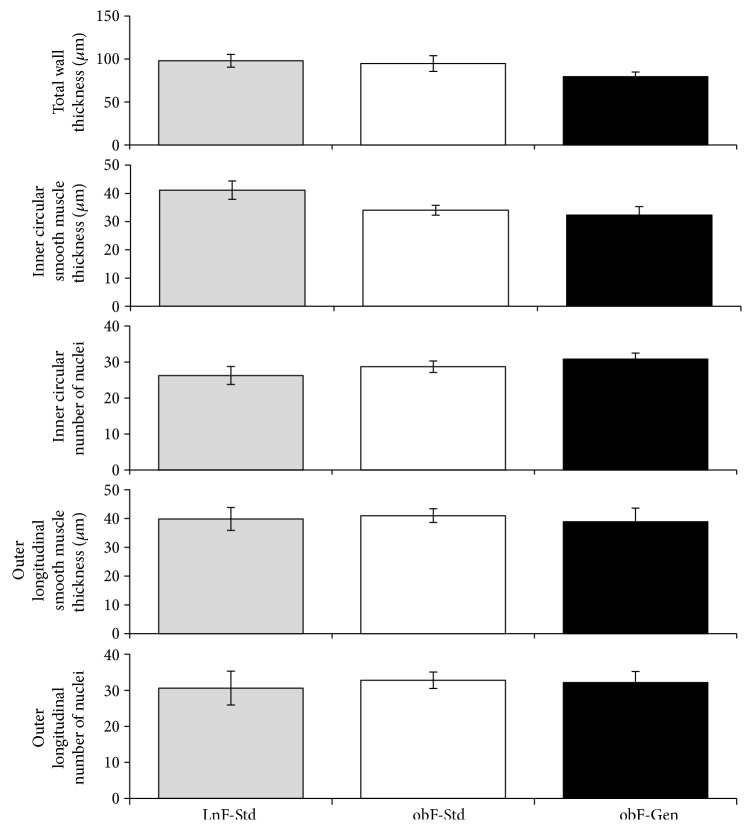
Effect of genistein on jejunum wall morphology. Total wall thickness. Lean mice fed standard diet (gray bar), *ob/ob* mice fed standard diet (open bar), and *ob/ob* mice fed genistein diet (solid bar). *n* = 12–17. Inner circular smooth muscle thickness. *n* = 6–9. Numbers of nuclei within the inner circular smooth muscle layer. *n* = 4–9. Outer longitudinal smooth muscle wall thickness. *n* = 6–9. Numbers of nuclei within the outer longitudinal smooth muscle layer. *n* = 4–9. Values are means ± SEM.

To determine whether jejunum morphology is modified by diabetes and to ascertain whether a genistein diet can modify wall thickness, histological sections were stained using H&E and analyzed for total wall thickness, circular smooth muscle thickness, longitudinal smooth muscle thickness, and numbers of nuclei per smooth muscle layer (Figure 2). There were no changes in any of the parameters measured.

### 3.3. Acetylcholine Receptors

**Figure 3:**
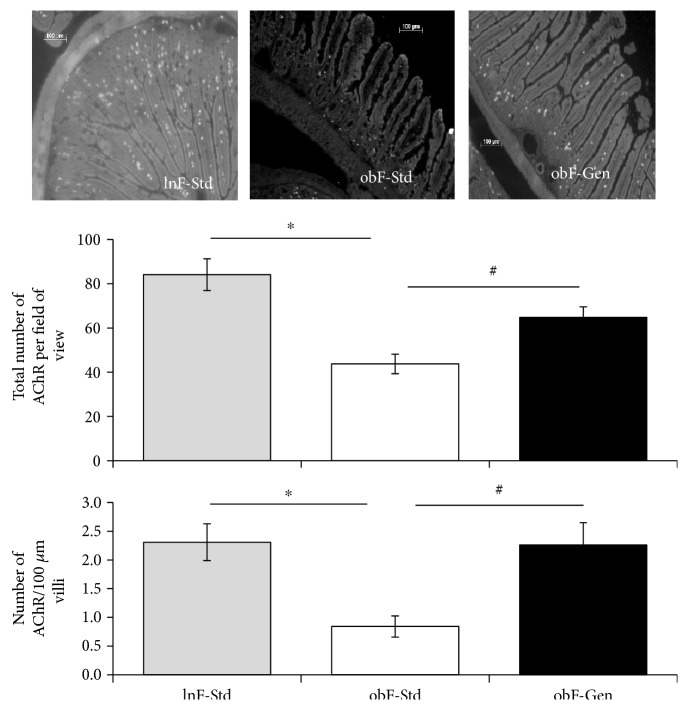
Effect of genistein on total numbers of acetylcholine receptors (AChR). Representative images of AChR clustering in females. Rhodamine-labeled AChR clusters were observed in lean female standard fed (LnF-Std), *ob/ob* female standard fed (obF-Std), and *ob/ob* female genistein fed (obF-Gen). Scale bar is 100 *μ*m. Average total number of AchRs per field of view and average AChR per 100 *μ*m length of villi. Lean mice fed standard diet (gray bar), *ob/ob* mice fed standard diet (open bar), and *ob/ob* mice fed genistein diet (solid bar). Values are means ± SEM (*n* = 6–13). ^∗^Significant difference from lean, *P* < 0.05. ^#^Significant genistein-mediated effect, *P* < 0.05.

AChRs were visualized and quantified in all groups of mice (Figure 3). AChRs were significantly decreased by 48% in the *ob/ob* female mice (43.75 ± 4.76, *n* = 13) compared to lean counterparts (84.10 ± 7.19, *n* = 10, *P* < 0.05, Figure 3). Interestingly, *ob/ob* mice fed a genistein diet had a 1.48-fold significant increase (64.82 ± 7.21, *n* = 13, *P* < 0.05) in the total number of AChRs compared to *ob/ob* female control counterparts fed a standard diet (43.75 ± 4.76^,^*n* = 13); that is, genistein diet partially, yet significantly, rescued the number of AChRs (Figure 3). In addition, we quantified the number of AChRs per 100 *μ*m length of villi to normalize for changes in villi length between groups: AChRs were significantly decreased in the *ob/ob* mice (0.84 ± 0.18, *n* = 13) compared to leans (2.31 ± 0.31, *n* = 13, *P* < 0.05, Figure 3), and the genistein diet induced a significant rescue of AChR clusters (2.26 ± 0.39, *n* = 13, *P* < 0.05, Figure 3).

### 3.4. Motility

**Figure 4:**
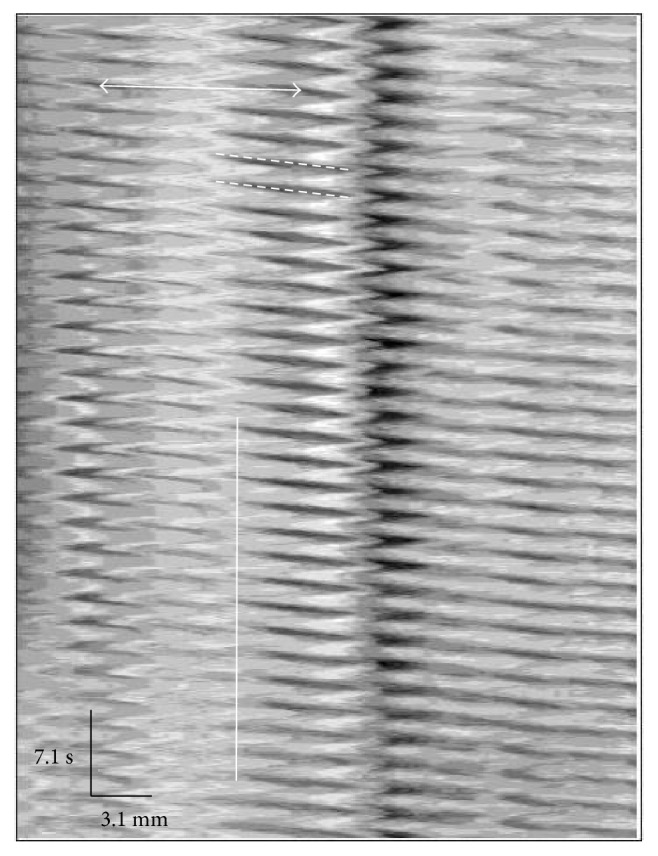
Representative trace from a video recording jejunum motility. A representative trace from lean jejunum. The following parameters are measured: velocity (slope of dashed white lines), total distance (length of white dashed line), and total time (based on length of white dashed line). To calculate the number of events occurring in a specific time period, calculate white solid vertical line duration, and divide by number of events in that time (15 events in this example). Distance between active regions is determined by drawing a line from the peak of one contraction wave to the peak of the subsequent wave (along a single row of the spatiotemporal map, see white solid line with arrow ends).

Motility characteristics were determined using the GIMM video capturing system. An example of a trace recorded using this system to evaluate motility is shown in Figure 4.

**Figure 5:**
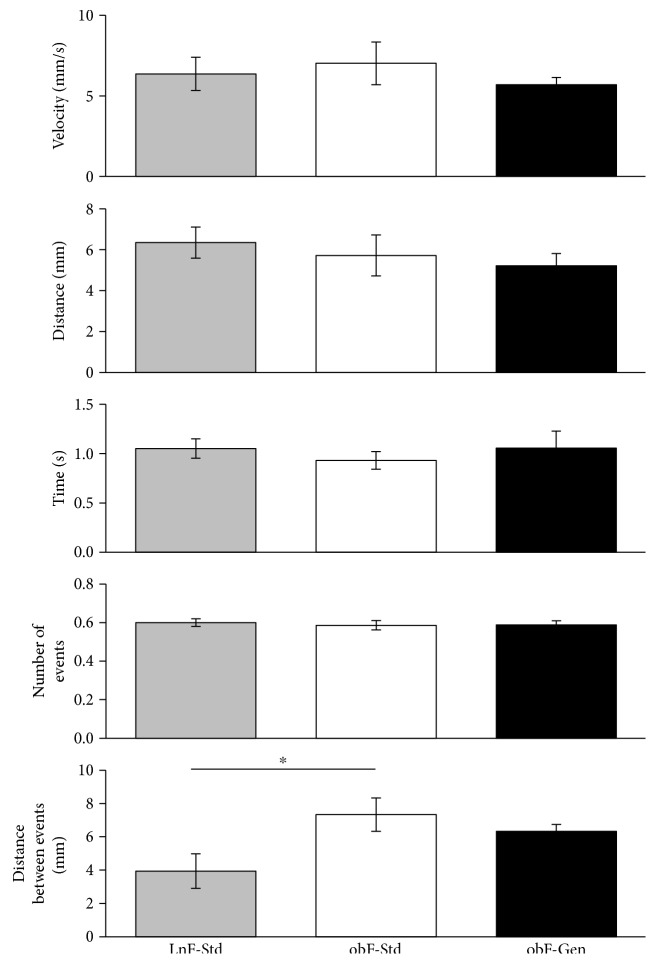
Effect of genistein on jejunum motility. Velocity (mm/s). Distance (mm). Time (s). Number of events. Distance between events (mm). Lean mice fed standard diet (gray bar), *ob/ob* mice fed standard diet (open bar), and *ob/ob* mice fed genistein diet (solid bar). Values are means ± SEM (*n* = 9 − 13). ^∗^Significant difference from lean, *P* < 0.05.

We found no effect of diabetes (or genistein) on velocity, distance, time, or number of events in isolated segments of the jejunum (Figure 5). However, we note a significant increase in the distance between events in a diabetic jejunum (7.33 ± 0.99, *n* = 6, *P* < 0.05) versus lean controls (3.95 ± 1.04, *n* = 7, Figure 5), with no genistein effect (6.32 ± 0.42, *n* = 7).

## 4. Discussion

The diabetic *ob/ob* mouse model is leptin deficient and hyperphagic, obese, and insulin resistant [16]. The advantage of this model of diabetes is the observation that intestinal difficulties that resemble those seen clinically in diabetes (i.e., slowed gastrointestinal transit [2] and delayed gastric emptying [3]) are also noted in the *ob/ob* model. The *ob/ob* mice used in this current study (12-13 weeks) are comparable to previous studies demonstrating altered intestinal function along with the typical symptoms of diabetes for this age range. In male *ob/ob* mice (aged 6–15 weeks) or male C57Bl/6J mice fed a high-fat diet for 14 weeks, gastroparesis has been demonstrated to be associated with twofold elevations in plasma glucose levels [6, 17]. Of note, 15-week-old male *ob/ob* mice have been shown to exhibit slowed gastrointestinal transit correlated to increased duodenal secretin content and decreased colonic vasoactive intestinal peptide content [2, 18]. In addition, Kiely et al. [19] have shown that 13-14-week-old male *ob/ob* mice exhibit slowed gastrointestinal transit time.

The pathogenesis of delayed diabetic gastrointestinal transit is not well understood, and current evidence in this field indicates a complex etiology. In diabetic db/db mice, disturbed gastrointestinal motility has been attributed to reduced areas of interstitial cells of Cajal [20]. Delayed gastrointestinal transit in obese diabetic mice has been associated with loss of duodenal secretin and colonic serotonin cell number [2]. The small intestine, specifically the jejunum, remains an understudied region in models of diabetes and obesity. Our results show significant changes in female *ob/ob* mouse jejunum that are consistent with dysfunctional intestinal transit: (1) reduced clusters of AChR and (2) increased distance between consecutive contractile events. These data combined suggest that structural changes and/or alterations in protein expression likely contribute towards an increased gastrointestinal transit time and thus contribute towards the diabetic phenotype of this model.

Transit of material through the gastrointestinal tract relies on a balanced release of contractile neuromodulators orad of the food bolus and the release of relaxative neuromodulators caudad of the bolus, thus propelling the food in a forward direction (proximal to distal). With regard to the involvement of acetylcholine receptors in the development of intestinal symptoms associated with diabetes, Liu et al. have shown that responsiveness to acetylcholine (and substance P) was significantly decreased in diabetic small intestine [21]. We are unaware of another study that has investigated the number of clusters of acetylcholine receptors in the jejunum of the *ob/ob* diabetic model. Therefore, our current data indicating a 48% loss of AChR clustering in *ob/ob* female mice (compared to leans, Figure 3) is intriguing and suggests that deficits in AChR could be responsible (at least in part) for the reduced gastrointestinal transit in this model. The genistein diet significantly improved the numbers of AChRs. Genistein is a known positive allosteric modulator of AChR. Acetylcholine is considered the main excitatory neuromodulator within the enteric nervous system. Interestingly, previous studies have shown that ligand-gated *α*7 nAChR is regulated through protein tyrosine kinase inhibitors such as genistein, which upregulates nAChRs expressed in *Xenopus* oocytes [22]; moreover, those data indicated that tyrosine phosphorylation regulates the distribution of these receptors via SNARE-dependent exocytosis into the plasma membrane. Whether or not the genistein-dependent increase in AChR clusters in *ob/ob* jejunum is mediated via SNARE-dependent exocytosis remains to be seen. Of note, genistein has been shown to have no effect on either the amount or distribution of AChR at the surface of SH-*α*7 cells (neuroblastoma cells stably transfected with human *α*7) [23]. Thus, genistein's mechanism of action and regulation of AChR is likely system or tissue specific. While our study has focused on potential variations in contractility of gastrointestinal smooth muscle, previous studies have purported that nonadrenergic noncholinergic relaxations are significantly reduced in a diabetic jejunum, associated with a significant loss of nNOS expression [24].

Our data suggests that while total wall thickness was not changed with diabetes, neither the thickness of the inner circular smooth muscle nor the outer longitudinal smooth muscle was changed (Figure 2). Moreover, the genistein diet did not alter nuclei number, indicating a lack of hyper- or hypotrophy of the smooth muscle cells in the walls of the gastrointestinal tract. Thus, in the *ob/ob* female mouse jejunum, physical modifications of smooth muscle could not explain neither the slowed intestinal transit of this diabetic model nor the beneficial effects of the genistein diet thereon. Interestingly, overall maximum contraction generated in isolated rings of jejunum was comparable between *ob/ob* females and lean counterparts (Figure 1()). However, in the absence of receptor-dependent contractility measures, with agonists such as acetylcholine, overarching and mechanistic conclusions cannot be made at this time.

The use of the gastrointestinal motility monitoring system (GIMM) affords us the ability to determine various parameters related to the intrinsic contractile nature of isolated segments of the jejunum. In this subset of experiments, we continuously analyzed motility for at least one minute of recording and evaluated the following (Figures 4 and 5): velocity, distance, time, number of events, and distance between events. To this point, the GIMM system has been effectively utilized to analyze propulsive motility through colonic tissue [25, 26]. We are unaware of other studies using this methodology to evaluate small intestinal motility in murine models. We believe applying this technique to mouse models of intestinal pathology will shed light on the involvement of both contracting and relaxing agents that result in intestinal dysmotility. Our future studies will be directed towards assessing pharmacological tools to reduce the distance between consecutive contractile events in *ob/ob* mouse intestines (i.e., returning towards lean levels).

The transit time through the intestine is associated with effective nutrient absorption throughout the intestine. The assumption that longer transit times are directly correlated with increased absorption and greater weight gain seems logical but is likely more complicated. Indeed, obese patients have been shown to have elevated absorption in the small intestine along with a shorter transit time [27]. This has been further verified in studies of isolated resected small intestine, wherein carbachol-induced smooth muscle contractility was shown to be increased and associated with the faster transit time [28]. Thus, a plethora of dysregulated pathways is likely involved in the presentation of intestinal dysfunction in the diabetic mouse model and in patients.

## 5. Conclusions

In conclusion, this study demonstrates two mechanisms that could be responsible for the delayed gastrointestinal transit in *ob/ob* murine jejunum. We propose that this dysfunction in intestinal transit is attributed to a combination of reduced numbers of AChRs and an increased distance between consecutive contractile events. This duo of incongruities likely contributes to the slowed gastrointestinal transit time, typical of the *ob/ob* mouse, and may also explain the human diabetic intestinal syndrome. Furthermore, we demonstrate that a genistein diet significantly recovers the numbers of AChRs bringing them closer to levels seen in lean controls, and while genistein diet does not significantly reduce distance between consecutive contractile events, there is certainly a trend to do so. Future studies will aim to better understand whether cellular regulators of AChR and/or other neuromodulators are also altered in *ob/ob* mice. We predict that the improvement of these intestinal dysfunctions might provide a method for therapeutic relief of the gastrointestinal complications seen in the diabetic phenotype.

## Abbreviations

AChR: Acetylcholine receptor
PBS: Phosphate-buffered saline
OCT: Optimal cutting temperature compound
H&E: Hematoxylin and eosin
GIMM: Gastrointestinal motility monitoring system.

## Data Access

The datasets used and/or analyzed during the current study are available from the corresponding author on reasonable request.
